# SUMO and cellular adaptive mechanisms

**DOI:** 10.1038/s12276-020-0457-2

**Published:** 2020-06-26

**Authors:** Hong-Yeoul Ryu, Seong Hoon Ahn, Mark Hochstrasser

**Affiliations:** 1 School of Life Sciences, BK21 Plus KNU Creative BioResearch Group, College of National Sciences, Kyungpook National University, Daegu, 41566 Republic of Korea; 2 Brain Science and Engineering Institute, Kyungpook National University, Daegu, 41566 Republic of Korea; 3 Department of Molecular and Life Science, College of Science and Convergence Technology, Hanyang University, Ansan, 15588 Republic of Korea; 4 Department of Molecular Biophysics and Biochemistry, Yale University, New Haven, CT 06520 USA

**Keywords:** Biochemistry, Molecular biology

## Abstract

The ubiquitin family member SUMO is a covalent regulator of proteins that functions in response to various stresses, and defects in SUMO-protein conjugation or deconjugation have been implicated in multiple diseases. The loss of the Ulp2 SUMO protease, which reverses SUMO-protein modifications, in the model eukaryote *Saccharomyces cerevisiae* is severely detrimental to cell fitness and has emerged as a useful model for studying how cells adapt to SUMO system dysfunction. Both short-term and long-term adaptive mechanisms are triggered depending on the length of time cells spend without this SUMO chain-cleaving enzyme. Such short-term adaptations include a highly specific multichromosome aneuploidy and large changes in ribosomal gene transcription. While aneuploid *ulp2Δ* cells survive, they suffer severe defects in growth and stress resistance. Over many generations, euploidy is restored, transcriptional programs are adjusted, and specific genetic changes that compensate for the loss of the SUMO protease are observed. These long-term adapted cells grow at normal rates with no detectable defects in stress resistance. In this review, we examine the connections between SUMO and cellular adaptive mechanisms more broadly.

## Stress response: how cells wrestle with SUMO disruption

Cellular stress caused by disrupting attachment of the ubiquitous small ubiquitin-like modifier (SUMO) proteins, which are present in most organisms and regulate numerous DNA processes and stress responses by attaching to key proteins, results in some remarkable adaptations. Mark Hochstrasser at Yale University, New Haven, USA, and co-workers review how this “sumoylation” is reversed by protease enzymes, and how imbalances between sumoylation and desumoylation may be linked to diseases including cancer. When certain SUMO proteases are deliberately disrupted, the cells quickly become aneuploid, i.e., carry an abnormal number of chromosomes. These cells show severe growth defects, but over many generations they regain the normal number of chromosomes. They also undergo genetic changes that promote alternative mechanisms that compensate for losing the SUMO protease and facilitate the same efficient stress responses as the original cells.

## Introduction

The small ubiquitin-like modifier (SUMO) protein is evolutionarily conserved and found in all eukaryotes^1^. While humans have four genes encoding SUMO proteins, SUMO1, 2, 3, and 4, the budding yeast *Saccharomyces cerevisiae* has only a single SUMO gene, *SMT3*, whose protein product shares 48% identity and 75% similarity with human SUMO1^2^. In the sumoylation pathway, SUMO is translated as a C-terminally extended precursor, which is subsequently trimmed by SUMO-specific proteases to release the mature form. The protein is conjugated to lysine side chains of target proteins via an enzyme cascade similar to that used for ubiquitin-protein conjugation. A heterodimeric SUMO-activating enzyme (E1) first forms a thioester linkage through its active-site cysteine with the carboxy terminus of SUMO, and the SUMO moiety is then transferred to the active-site cysteine of a SUMO-conjugating enzyme (E2). Typically, SUMO is then conjugated to a lysine side chain(s) of a substrate protein, which is mediated by one of the several SUMO ligases (E3s)^3^. Chains of SUMO can also assemble on substrates. Sumoylation is reversed by site-specific proteases; nine SUMO proteases have been reported in humans and two, Ulp1 and Ulp2, in *S. cerevisiae*^4^.

The SUMO protein posttranslationally modifies diverse substrates involved in various cellular processes, including transcription, DNA replication, cell-cycle progression, nucleo-cytoplasmic transport, apoptosis, and genome integrity and stability^5^. SUMO is an essential regulator of cell homeostasis when cells encounter environmental stresses such as osmotic shock, hypoxia, heat, oxidative stress, nutrient deprivation, or genotoxic stresses, and protein sumoylation levels increase sharply in response to stress^6^. Although the SUMO stress response is still not fully understood, it was previously reported that the Siz1 E3 ligase and Ulp2 SUMO protease are major factors involved in the SUMO stress response in *S. cerevisiae*^7^.

Recently, our group reported that distinct adaptive mechanisms counter a dysregulated SUMO system upon loss of the Ulp2 protease^8,9^. To overcome the stress caused by the acute loss of Ulp2, mutant yeast cells become aneuploid (i.e., they carry an abnormal number of chromosomes), which promotes compensatory mechanisms for rapid adaptation to Ulp2 loss. However, because aneuploidy is usually deleterious to cell fitness^10^ and such *ulp2Δ* cells exhibit severely impaired growth, long-term adaptation restores euploidy and leads to countervailing mutations in SUMO conjugation enzymes and regulatory shifts in ribosome biogenesis. The stepwise employment of these mechanisms in response to disturbed SUMO conjugation dynamics is likely relevant to the robust adaptive fitness gains of cells following the loss of the quasi-essential Ulp2 protease. The present review provides an overview of the mechanisms of adaptation to environmental stress and particularly how the perturbation of the SUMO system is countered.

## First-line adaptive mechanisms

The types of adaptation that occur in response to exogeneous or endogenous stress stimuli generally depend on the severity, duration, and reversibility of the stress conditions. If they do not exceed a certain threshold, stress effects are counterbalanced by transient protective mechanisms that promote cell survival^11^. Rapid “first-line” cellular responses include changes in metabolism, gene expression, cell-cycle progression, protein homeostasis, cytoskeletal organization, vesicular trafficking, and/or enzyme activity, which can re-establish homeostasis and maintain viability. For example, many heat shock proteins function as molecular chaperones that ensure the proper refolding of misfolded proteins and prevent or reverse protein aggregation under multiple environmental stress conditions^12^. Another example is the unfolded protein response, a highly conserved eukaryotic signaling pathway that responds to the accumulation of unfolded or misfolded proteins in the endoplasmic reticulum (ER)^13^. The accumulation of such aberrant proteins is sensed by transmembrane proteins in the ER that activate specific transcriptional programs; these include genes for ER molecular chaperones and ER-associated degradation pathways to help clear the ER of damaged proteins. In many species, protein translation is also decreased, and ER-associated mRNAs are selectively destroyed to reduce the protein client load of the ER protein folding machinery.

**Fig. 1:**
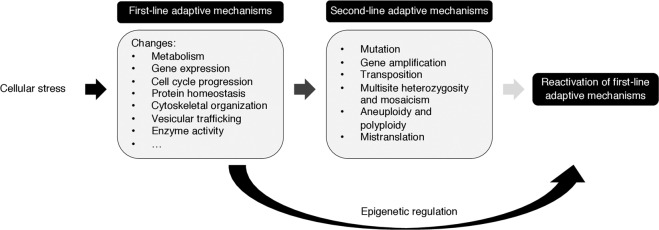
Stress-induced cellular adaptive strategies. First-line adaptive mechanisms maximize cell survival under acute stress conditions. When these protective responses are not sufficient to protect cells from stress, the cells activate second-line adaptive mechanisms, which primarily consist of genetic changes that confer resistance to stress. However, because some second-line responses are deleterious to cell fitness, they may evolve or reactivate other adaptive mechanisms through genetic or epigenetic changes.

Other examples include disturbances in the balance between pro- and antioxidant factors, which cause an oxidative stress response that upregulates many antioxidant genes^14^, and DNA double-strand breaks (DSBs) and single-strand breaks induced by genotoxic stressors, which require the DNA lesions to be detected and repaired by the DNA damage response^15^. All of these first-line protective responses involve stresses that are reversible or short term. If a stress is persistent or irreversible, cells often engage a set of genetic, “second-line” adaptive mechanisms, which allow them to survive in the continued presence of the stress (Fig. 1)^16^.

## Second-line adaptive mechanisms

### Mutation

Unlike spontaneous mutations, so-called adaptive mutations are genetic variations that are apparently induced specifically by exposure to an environment in which the mutation provides a selective advantage^17^. This contrasts with natural selection, in which cells bearing pre-existing genetic variants are selected if these mutations provide a reproductive advantage. Most studies of directed adaptive mutation, which remains a highly controversial concept (for example, see ref. ^18^), have been performed in *Escherichia coli*, but additional studies have been reported in other bacteria and eukaryotes, including yeast and mammals^17,19,20^. While there is no consensus regarding whether directed mutation can occur, there is a range of reported evidence for stress-induced hypermutation, which can increase the probability of creating favorable mutations that can be acted upon by natural selection^21^.

Stress-induced mutations include base substitutions, deletions, and insertions, and they are generated by evolutionarily conserved mechanisms. For instance, high rates of transcription have been associated with higher mutation frequencies due to exceeding the capacity of transcription-coupled DNA repair, which normally represses elevated rates of recombination and mutagenesis during transcription in bacteria and yeast^22–24^. The accumulation of genetic alterations in certain liver cancer cells also depends on active transcription^25^. Error-prone DNA polymerases are responsible for stress-induced mutations in bacterial *lac*, yeast *CAN1*, and human androgen receptor genes^26–28^. On the other hand, defects or limitation of DNA-repair processes caused by particular stress conditions in bacteria and mammalian cells or aneuploidy stress in yeast leads to increased mutation rates^29–31^. There is a switch from homologous recombination-mediated DSB repair to the more error-prone nonhomologous end-joining DNA-repair pathway under specific stress conditions in yeast^32^, and similar mechanisms have been reported in human cancer cells^33^.

### Gene amplification

A study involving the Cairns and Foster *lac* frameshift system revealed that gene amplification could also contribute to Lac+ colonies following long periods of incubation on lactose medium^34^. Because the DNA junctions of amplified units are located in regions including nonhomologous joints in Lac^+^ cells^34^, a plausible hypothesis is that adaptive point mutations and gene amplification are both commonly initiated from DSBs, but amplification might primarily occur when no homologous sequence is available during repair. In yeast, long-term adaption to a glucose-limited environment also leads to the amplification of genes encoding the high-affinity hexose transporter^35^, and in human glioblastomas, double minutes, another manifestation of gene amplification, develop in response to hypoxia, a tumor microenvironment factor^36^.

### Transposition

Transposons are DNA elements that autonomously move to distal locations within the genome, often inducing or reverting existing mutations^37^. Transposon mobility contributes to diverse regulatory mechanisms^38^. For example, glycerol limitation triggers the site-specific insertion of the IS5 transposon into a site upstream of the *E. coli glpFK* operon^39^, activating the expression of the operon to promote glycerol utilization. Similarly, in glucose-limited yeast cultures, specific chromosomal rearrangements may bring an activating transposon sequence to a position next to the *CIT1* gene, which encodes citrate synthase, a key enzyme in the tricarboxylic acid cycle^35^. In human neuroblastoma cells, oxidative stress induces an increase in the retrotransposition of long interspersed element-1 and the upregulation of its transcript^40^.

### Multisite heterozygosity and mosaicism

Multisite heterozygosity is defined as the coexistence of differences in the genetic composition at multiple loci in the same population and is often increased by outcrossing between different populations under environmental stress conditions^41^. Outcrossing between two or more populations gives rise to a larger proportion of allelic variation or mosaicism in the genome. The DNA sequencing of natural isolates of yeast strains, which tend to preferentially undergo asexual reproduction, revealed ~63% heterozygosity, suggesting some fitness advantage for this condition^42^. Higher levels of heterozygosity in yeast are observed under diverse stress conditions, such as exposure to the antifungal drug fluconazole, growth at high temperature, or incompatibility of the mismatch repair genes *MLH1-PMS1*^43,44^.

### Aneuploidy and polyploidy

Aneuploidy (an abnormal number of chromosomes) is usually acquired through unequal chromosome segregation during cell division, resulting in an imbalance in chromosome copy number, accompanied by parallel changes in both mRNA and protein levels^10,45^. An extra or missing chromosome has detrimental effects on cellular fitness and causes genomic instability in various organisms, including *Drosophila*, *C. elegans*, mice, plants, and humans^46^. In addition, almost all cancer cells, including 90% of solid tumors and 75% of hematopoietic cancers, have an aberrant number of chromosomes^47^; however, it is still debated whether such aneuploidies are a cause or consequence of cancer.

Although aneuploidy generally reduces cell fitness, it can provide a selective advantage relative to euploid cells under certain circumstances^10^. In the case of such beneficial aneuploidy, the increased dosage of a specific gene(s) on the duplicated chromosome(s) can mitigate the deleterious effects of the stress that induced the aneuploidy. Yeast has emerged as a versatile model organism for studying the adaptive effects of aneuploidy^48^. Adaptive aneuploidies are triggered in response to various stresses, including the application of the drugs fluconazole, radicicol, and 4-NQO; the deletion of genes such as *MYO1*, *RPS24A*, or *RNR1*; nutrient limitation; high temperature; or high pH. Since aneuploidy is an acute compensation mechanism with long-term fitness costs, continued encounters with the same stress often activate alternative adaptive mechanisms that can restore euploidy^49^. For example, specific aneuploidies induced by heat or high pH have been found to be eliminated and replaced with (unidentified) gene mutations and alterations in gene expression after long-term exposure to these conditions^50^.

Adaptive polyploidy, usually resulting from whole-genome duplication, has been reported in some cases. In vitro laboratory evolution experiments with baker’s yeast revealed that tetraploids experience accelerated adaptation compared with haploids and diploids^51^. In the pathogenic yeast *Cryptococcus neoformans* grown in the presence of antifungal azole drugs, transient polyploid states appear to give rise to aneuploid progeny with heightened drug resistance; this situation increases the dosage of specific genes, including *ERG11*, which encodes the azole drug target^52^. The treatment of human HL-60 cells with SKF 10496, which targets the *ERG11* homolog that functions in cholesterol biosynthesis, has been found to lead to polyploidy^53^. Therefore, both polyploidy and aneuploidy can provide selective advantages under specific stress conditions.

### Mis-translation

An increased translation error rate is a less established mechanism for adaptation to cell stress. The fidelity of translation is normally quite high, at approximately one error per 10^3^–10^4^ amino acids incorporated into proteins; fidelity is maintained through highly accurate tRNA aminoacylation and mRNA codon-cognate tRNA anticodon pairing by the ribosome^54^. The error rate of protein synthesis is typically increased under stressful conditions, which provoke tRNA misacylation, ribosome miscoding, frameshift errors, and translational readthrough, but cells can tolerate substantial decreases in translation fidelity^55^. Although the synthesis of mutant proteins frequently leads to protein misfolding or aggregation, some mutant protein forms can enhance specific stress responses and adaptations^55^. For instance, misfolded proteins are preferentially directed to the proteasome, and the peptide fragments generated by proteasomes can serve as ligands in antigen presentation^56^. An intriguing model has been proposed in which methionine-substituted mutant proteins resulting from tRNA misacylation with Met during oxidative stress could potentially provide sites for reversible modification by reactive oxygen species, sparing oxidizable active-site residues in these proteins^55^. Met-enriched proteins tend to remain active longer than the equivalent Met-depleted versions under oxidative stress conditions^57,58^. In the human commensal yeast *Candida albicans*, translation of CUG codons as either Ser or Leu can result in greater diversity in cell surface proteins that are normally recognized by the host immune system or can lead to resistance to the antifungal agent fluconazole^59,60^.

## Return to first-line or other adaptive mechanisms

As noted above, most first-line responses to stress stimuli are rapidly reversible when the stress is removed or ameliorated, allowing the cell to return to its basal state^16^. When exposed to persistent stresses, second-line defense mechanisms are triggered, but cells often reactivate first-line mechanisms that impose a lower long-term fitness cost (Fig. 1). A clear example was reported by Dahan and colleagues, who showed that the aneuploidy-based adaptation of yeast to high temperature was replaced by refinements in gene expression during prolonged evolution at 39 °C^50^.

Epigenetic regulation appears to play a critical role in these adjustments of the transcriptional program. Such epigenetic mechanisms allow rapid and reversible, but durable adaptations through histone or DNA modifications that mediate changes in transcription, chromatin structure, or pre-mRNA processing^61^. Prion-mediated changes in protein states, which can be inherited without changes at the genetic level, may provide another mechanism of adaptation^62,63^. Cells can therefore switch or combine distinct adaptive strategies to maximize survival under various selective pressures.

## SUMO and stress responses

SUMO is an essential modulator of cellular responses to various environmental stresses, such as heat, oxidative, osmotic, or genotoxic stresses, which lead to increased global levels of sumoylation in yeast and mammals^6^. The sumoylation level of target protein(s) depends on the nature, duration, and intensity of the stress^64^. The conjugation of SUMO to individual targets can be dramatically altered in response to specific stresses. For instance, heat shock induces a large increase in the SUMO conjugation of targets such as the heat shock factor-1 (HSF1) and c-Myb transcription factors^65,66^, whereas the sumoylation of the c-Fos, topoisomerase 1, and promyelocytic leukemia proteins decreases upon heat shock^67–69^. An interesting response is observed under different levels of oxidative stress. Low doses of H_2_O_2_ (below 10 μM) have minimal effects on SUMO conjugation, but moderate amounts of H_2_O_2_ (below 1 mM) lead to the inhibition of global sumoylation due to the formation of disulfide bond(s) between the catalytic cysteines of the SUMO E1 and E2 enzymes^70–72^. High doses of oxidants actually increase bulk SUMO-2/3 conjugate levels, probably through the attenuation of SUMO protease activity. Hypoxic stress induces increased global protein sumoylation through the upregulation of the expression of SUMO1^61,73^. SUMO is crucial for the response to genotoxic stress as well, and the inhibition of the SUMO pathway leads to increased sensitivity to a wide range of genotoxic agents in both yeast and human cells^74,75^.

A key element in the SUMO stress response is the regulation of transcription. SUMO suppresses the transcriptional activity of diverse activators, including c-Myb, a major regulator of cell proliferation^65^. Conversely, transcription can be activated by the sumoylation of proteins such as HSF1, resulting in increased DNA binding and activity^66^, or NEMO, which activates NFκB in response to genotoxic stress^76^. Other targets of the SUMO stress response include basal components of the transcription machinery such as the TFIID and Mediator complexes, chromatin remodeling factors, the transcriptional corepressor Tup1-Cyc8, and subunits of the Set3 and Rpd3 histone deacetylase complexes^7^. A common feature of various types of stress is the desumoylation of RNA polymerase III subunits, which correlates with a decrease in tRNA transcription^77^. Histone sumoylation is also generally associated with transcriptional repression^78^, and its removal by the Ulp2 SUMO protease is required for the promotion of RNA polymerase II transcription elongation in yeast^79^.

## The Ulp2 SUMO protease and adaptations to its loss

**Fig. 2:**
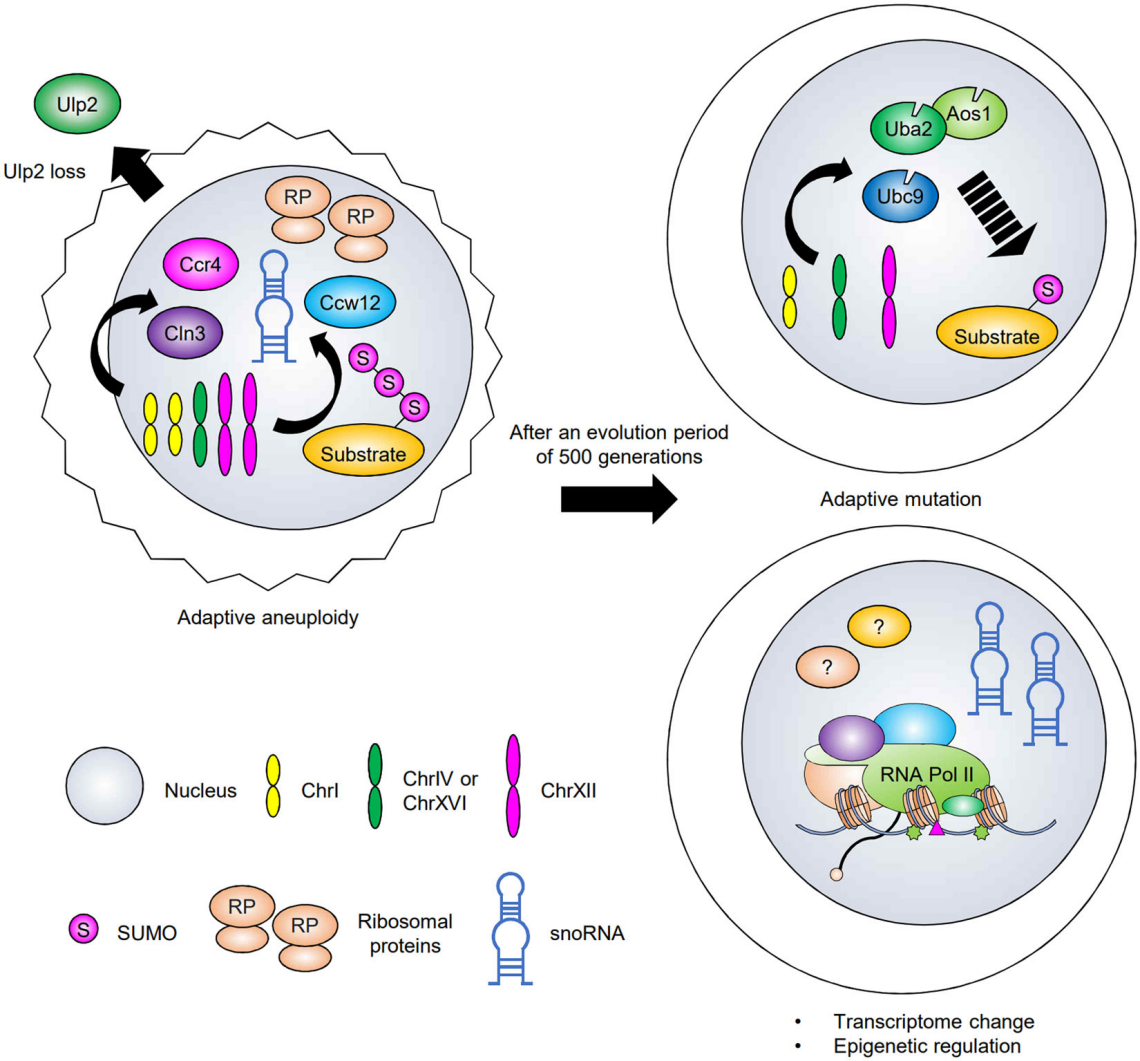
Evolution of adaptive mechanisms upon the loss of Ulp2. The loss of Ulp2 in yeast leads to the accumulation of polySUMO-conjugated proteins, increased expression of ribosomal proteins and reduced cell fitness (depicted by the irregular cell outline). Disomies of ChrI and ChrXII provide a transient adaptive solution by virtue of an increased dosage of three protein-coding genes, *CCR4*, *CLN3*, and *CCW12*, and a snoRNA gene cluster consisting of *SNR61*, *SNR55*, and *SNR57*. Following evolution over many cell generations, disomies of both ChrI and XII are replaced with two other adaptive mechanisms: mutations of SUMO-ligating enzymes and specific transcriptome changes. Point mutations in *UBC9* or *UBA2*, on ChrIV, or *AOS1*, on ChrXVI, reduce SUMO conjugation and suppress the growth defects of *ulp2Δ* cells. In parallel, the upregulation of numerous snoRNA genes, which can repress the transcription of RP genes, and refined transcriptome alterations concomitant with epigenetic changes (unpublished data) appear to facilitate further adaptation.

Although the SUMO system is implicated in the regulation of many cellular processes, how cells adapt to inhibition or dysfunction of the system is still largely unknown. As a first step in analyzing such mechanisms, our group examined how cells adapt to the loss of the yeast Ulp2 SUMO protease, a polySUMO chain-depolymerizing enzyme (Fig. 2). The deletion of the *ULP2* gene causes severe growth defects even under optimal conditions^80^. Whole-genome RNA sequencing revealed that cells that survived the elimination of Ulp2 display a twofold increase in transcript levels across two specific chromosomes, chromosome I and ChrXII; this was traced to the duplication of these chromosomes^9^. Ulp2 plays roles in chromosome segregation and the maintenance of centromere cohesion^81^, so its loss may also facilitate the generation of cells with this very specific but aberrant chromosome complement by increasing chromosome segregation errors.

The two-chromosome aneuploidy in *ulp2Δ* cells is an essential adaptation due to the increased dosage of three specific protein-coding genes, *CCR4*, *CLN3*, and *CCW12*, and a cluster of small nucleolar RNA (snoRNA) genes, *SNR61*, *SNR55*, and *SNR57*^8,9^, which are all carried by these two chromosomes. Increased levels of Ccr4, a catalytic deadenylase subunit of the Ccr4–Not complex, in *ulp2Δ* mutant cells limit the expression of snoRNA and ribosomal RNA (rRNA) and thereby controls the synthesis of ribosomes, which is usually tightly coupled to growth. This compensates for the absence of Ulp2, which would normally check ribosome levels by inhibiting the transcription of the ribosomal protein (RP), snoRNA, and rRNA genes^8^. Increased levels of the three snoRNAs expressed from ChrXII also reduce the transcription of RP genes; interestingly, a previous report showed that a multiple myeloma-associated snoRNA, ACA11, downregulates RP gene expression in human cells^82^. An increased dosage of *CLN3*, encoding a G_1_ cyclin, likely prevents the development of aneuploidy by promoting cell-cycle progression. The adaptive role of elevated Ccw12, a mannoprotein required for cell wall integrity, is unknown.

Although these aneuploidy-mediated compensatory mechanisms help to overcome the acute stress caused by a dysregulated SUMO system, aneuploidy is deleterious, and aneuploid *ulp2Δ* cultures grow poorly and are highly sensitive to many stresses^50,83^. However, in vitro evolution over 250–500 generations restores euploidy^8^. Remarkably, despite the complete absence of the *ULP2* gene, these evolved cells exhibit nearly normal growth and cell-cycle characteristics and do not present any obvious stress sensitivities. The evaluation of independent *ulp2Δ* cultures that evolved in parallel showed that the strains accrue different mutations in the genes encoding the SUMO-ligating enzymes Uba2/Aos1 (either subunit) or Ubc9. This is accompanied by a reduction in polySUMO-conjugate accumulation in most of the isolates. These results indicate that partial loss-of-function mutations in the essential SUMO ligation pathway can counter the hypersumoylation phenotype caused by Ulp2 loss^8^ and might affect the gene expression profile in a direction that increases cell fitness.

Interestingly, several of the evolved strains continued to maintain high levels of polySUMO conjugates, and no additional mutations in the SUMO pathway were found in these cells^8^. This indicates that additional adaptive mechanisms are possible. One such potential alternative mechanism might be the upregulation of snoRNA expression, which can repress the transcription of RPs and may help to resolve chromosome imbalances and restore stress resistance in *ulp2Δ* cells. However, additional copies of single snoRNAs do not suppress the growth defects caused by the loss of Ulp2^8^. In addition to the evolutionary trajectories described above, we recently identified altered transcriptional profiles of certain genes in evolved *ulp2Δ* cells and likely epigenetic changes; we are now analyzing their potential adaptive advantages (unpublished data).

Eukaryotic ribosome biogenesis is critically linked to the SUMO pathway. The mammalian SUMO protease SENP3 physically interacts with multiple proteins involved in ribosome maturation, including NPM1 and the PELP1-WDR18-TEX10 complex, which are required for rRNA processing and the transit of the 60S ribosomal subunit from the nucleolus^84^. Several SUMO pathway mutants have been shown to exhibit defects in rRNA processing in *S. cerevisiae* as well^85^. SUMO and the Ulp2 protease localize to RP genes, reflecting the regulation of RP gene expression by balancing the sumoylation and desumoylation of the Rap1 transcription factor^8,79,86^. Consistent with these close ties between protein sumoylation and ribosome formation, regulatory shifts in ribosome biogenesis are an important factor in the response of cells to Ulp2 loss^8^.

## Concluding remarks

Although SUMO is known to play essential roles in various stress responses, the adaptive mechanisms that allow cells to survive and flourish when elements of the essential SUMO system are altered have not been extensively explored. Here, we have briefly summarized various genetic or second-line adaptive responses to severe physiological stress or environmental insult, and we have described recent observations of the adaptation of cells to SUMO system dysfunction in particular. When the Ulp2 SUMO protease is lost, a complex adaptive aneuploidy is rapidly established; over longer periods, normal growth is usually restored by compensatory mutations in SUMO-ligating enzymes and the restoration of euploidy^8,9^. In response to severe or chronic stress, progression from rapid but nonideal adaptations to different, longer-term changes that boost fitness is likely to occur in association with many other pathways^50^. For instance, similar dynamic evolutionary trajectories may characterize a large fraction of tumors^47^.

The SUMO system is phylogenetically well conserved in eukaryotes, although it is not essential for viability in all organisms^87^. An interesting case relevant to the evolution of the SUMO system involves microsporidial *Encephalitozoon* spp. These obligate intracellular parasites have undergone extreme genome reduction, and their genome does not appear to encode a *ULP2* ortholog, although it still harbors genes encoding a rudimentary SUMO pathway based on available genome sequences^88^. These organisms might have experienced a parallel deterioration in SUMO conjugation and deconjugation activities to maintain an optimal balance between them, similar to what we observed in our in vitro evolution studies in *ulp2Δ* cells. Analogous high-throughput in vitro evolution experiments hold promise for deciphering other aspects of SUMO system physiology.

Imbalances between sumoylation and desumoylation have been suggested to be important in the development of multiple diseases, such as cancer and neurodegenerative disorders^89^. Several SUMO enzymes appear to be upregulated in different cancers, where they seem to protect the stability and functionality of gene expression programs and signaling pathways in the face of cancer-induced changes^90^. Conversely, protein sumoylation contributes to certain pathological conditions and neurological disorders by promoting the formation of toxic protein aggregates^89^. The study of SUMO function in adaptive mechanisms may provide clues for the development of new therapeutic agents for these different disorders.
